# A multi-objective optimization using response surface model coupled with particle swarm algorithm on FSW process parameters

**DOI:** 10.1038/s41598-022-06652-3

**Published:** 2022-02-18

**Authors:** Parviz Kahhal, Mohsen Ghasemi, Mohammad Kashfi, Hossein Ghorbani-Menghari, Ji Hoon Kim

**Affiliations:** 1 School of Mechanical Engineering, Pusan National University, 2 Busandaehak-ro 63beon-gil, Geomjeong-gu, Busan, 46241 South Korea; 2 Department of Mechanical Engineering, Ayatollah Boroujerdi University, 69199-69737 Boroujerd, Iran; 3 Mechanical Engineering Department, Dezful Branch, Islamic Azad University, Dezful, Iran

**Keywords:** Mechanical engineering, Mechanical properties

## Abstract

In this study, multi-objective optimization of mechanical properties in friction-stir-welding of AH12 1050 aluminum alloy is performed using a combination of the response surface method and multi-objective particle swarm optimization algorithm. The process parameters are considered as tool pin diameter, shoulder diameter, rotational speed, feed speed, and tool tilt angle. The heat-affected zone’s yield strength, fracture strain, impact toughness, and hardness on the advancing and retreating sides are selected as the objective functions. Threaded and simple conical pins are utilized to evaluate the effect of the pin geometry on the specimen mechanical properties. Optimization model outputs are in agree with the obtained experimental results. The effects of process parameters on the mechanical properties of the friction-stir-welded sheets are studied. Results reveal that the lower rotational speed and higher feed speed improve the material strength and hardness. Moreover, the microstructural analysis demonstrates that the proposed methodology can achieve a fine-grained structure with the minimum defects. Improvement in the material flow is observed for the threaded cylindrical pin compared with the conical pin due to the geometric shape of the tool pin leading to more functional mechanical properties. It is found that the combination of the response surface methodology and the multi-objective particle swarm algorithm led to the modeling and optimization of the process with outstanding accuracy and low experimental cost.

## Introduction

**Figure 1:**
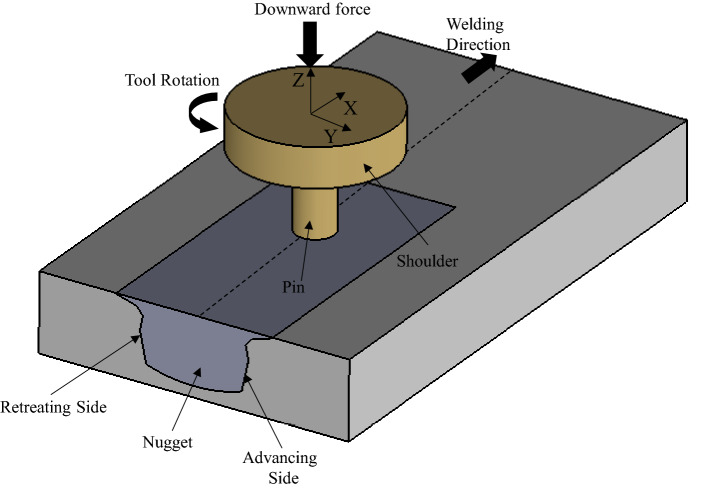
The FSW process nomenclature.

Friction stir welding (FSW), which involves making the joint without melting the parts, was introduced in 1991 by The Welding Institute (TWI)^1^. As shown in Fig. 1, two pieces of sheet metal are placed close to each other. A rotating tool is then placed at the joint, and welding is performed by moving the tool towards the joint^2^. Since the welding process in FSW is simultaneously performed by rotational and linear motions, the formed distortion and residual stress are reduced compared to the conventional welding processes. As the FSW process does not contain melted metal, the temperature of this process is lower than fusion welding, which reduces the thermal gradient in the welding zone and improves the mechanical properties and welding quality^1,3^.

Several studies have been conducted on FSW technology for similar and dissimilar alloys. Sahin^4^ investigated the parameters affecting the FSW of high-speed and medium–carbon steel. After finding the optimum welding parameters, the strengths of the joints have been determined by mechanical tests as well as hardness variations and microstructures examination. They compared the results with the strengths of the base materials. Leon and Jayakumar^5^ studied the effect of process parameters on the tensile strength, microstructure, and stiffness in the FSW of aluminum 6061. The changes in mechanical properties have been compared with the base metal. The key role of the tool rotational speed and welding speed on joint characteristics has been revealed. They reported that the tensile properties and impact strength of the FSW joints have been improved duo to the higher hardness and fine microstructure. Yousef et al.^6^ utilized an artificial neural network (ANN) to model the FSW parameters on the mechanical properties of aluminum sheets. They combined the influence of feed speed and pin rotational speed on the mechanical properties of welded aluminum sheets and achieved a good performance of ANN model. Sandaram and Muragan^7^ investigated the effect of pin geometry in the FSW of 2024 and 5083 aluminum alloys. They used five different tool pin profiles and four process parameters to create a predictive response surface model for ultimate tensile strength and tensile elongation. Results revealed that joints fabricated using a tapered hexagon tool pin profile achieve the highest tensile strength and tensile elongation. In contrast, the straight cylinder tool pin profile gives the lowest tensile strength and tensile elongation. Ghaffarpour et al.^8^ analyzed the effect of FSW process parameters on the 5083 and 6061 aluminum alloys joint mechanical properties. By using the response surface design methodology, they optimized the maximum tensile strength of the welding process and compared the results with those of the experimental test. Moreover, they studied the formability of welded sheets by using the limit dome height test. Yuvaraj et al.^9^ studied the effect of FSW process parameters on the tensile strength using the desirability function approach. The FSW parameters such as tool offset, pin profile and tilt angle have been considered for the experiments. Based on the ANOVA analysis, they revealed that the tilt angle of the tool has been the most controlling factor for improving the tensile properties of the joint, followed by tool pin profile and tool offset. Marimuthu and Pandiyarajan^10^ optimized the FSW process using four effective parameters and two objective functions. Optimization was performed using the desirability function approach and examining more efficient parameters in the process. The Box-Behnken three levels and three factors have been used to classify the number of experiments. Jangra et al.^11^ investigated rotational and feed speeds, tool pin profile, and tool shoulder diameter. They conducted several experiments on two similar sets, AA 6082-T6 and cryogenic treated AA6082 to compare the optimization results. A combined approach of Taguchi method, grey relational analysis, and entropy measurement method have been developed to find an optimal single setting of process parameters for two response characteristics. By examining four effective parameters, Verma et al.^12^ optimized the ultimate tensile strength and elongation percentage in aluminum alloy friction welding using genetic multi-objective optimization algorithm (MOGA) and hybrid genetic multi-objective optimization algorithm (HMOGA). Rotational speed, welding speed, and tilt angle have been used as input variables. They found that HMOGA provided better results than MOGA. In the aforementioned study, multi-objective optimization was performed using statistical methods, such as the desirability function.

Most problems in nature have several (possibly conflicting) objectives to be satisfied. Some of the nature-inspired optimization algorithms have been used frequently in engineering optimization problems^13^, including genetic algorithm (GA), ant colony optimization (ACO), particle swarm optimization (PSO), and artificial bee colony (ABC). The complexity of GA is more than PSO in principle for the same work. ACO is a time-consuming method, and the convergence time is also uncertain. ABC has a slow convergence rate, easy to fall in local optimum, and is difficult to find the best out of available feasible solutions. PSO is widely employed to solve the continuous problems because of the simplicity of concept and fewer parametric settings than other population-based optimization algorithms^14,15^.

Although, in the mentioned studies up to two objective functions are considered, in the present study, the FSW process of 1050 A-H12 aluminum is optimized using five objective functions and five influential design parameters. In addition, a multi-objective particle swarm optimization algorithm (MOPSO) is utilized known as a metaheuristic algorithm. The high convergence speed and relative simplicity of PSO make it a perfect candidate for the multi-objective optimization problems. The five objectives for optimization are considered as yield strength, impact toughness, failure strain, and hardness of the heat-affected zone on the advancing and retreating sides. The process parameters include pin diameter, shoulder diameter, rotational speed, feed speed, and tool tilt angle. After preparing test samples, tensile, impact, and hardness tests are conducted. A relation between the parameters and objective functions is estimated using the available data and the response surface method. Finally, the optimal solutions are obtained, and the predictions are compared with the experiments.

## Methods

### Multi-objective optimization procedure

#### Design of experiments: response surface model

The response surface method (RSM) is an approach for building approximations of objectives based on observations in the design space. This approach is functional when gradient-based methods fail^16^. The choice of surrogate functions to estimate the actual performance is crucial. These functions can be defined as polynomials or sums of various basis functions (e.g., sine and cosine).

This study employs a second-order polynomial to construct the response surface model. If *n*_*s*_ analyses are conducted and p = 1; 2; …; *n*_s_, then a second-order polynomial model has the following form

1 $$y^{\left( p \right)} = c_{o} + \mathop \sum \limits_{{1 \le j \le n_{v} }} c_{j} x_{j}^{\left( p \right)} + \mathop \sum \limits_{{1 \le j \le k \le n_{v} }} c_{{\left( {n_{v} - 1 + j + k} \right)}} x_{j}^{\left( p \right)} X_{k}^{\left( p \right)}$$

where *y*^(*p*)^ is the response, $x_{j}^{\left( p \right)}$ and $X_{k}^{\left( p \right)}$ are the *n*_*v*_ design parameters, and *c*_*o*_; *c*_*j*_; and $c_{{\left( {n_{v} - 1 + j + k} \right) }}$ are the problem coefficients^17^. Taguchi method is used to design the experiment, and a quadratic model is defined to construct the response surface model. A quadratic relation is obtained for each objective function, as shown in Eq. (2).

2 $$Obj = A\left( {PD} \right) + B\left( {SHD} \right) + C\left( F \right) + D\left( S \right) + E\left( {TA} \right) + G\left( {PD} \right)^{2} + H\left( {SHD} \right)^{2} + I\left( F \right)^{2} + J\left( S \right)^{2} + K\left( {TA} \right)^{2} + L$$

where **PD** is the tool pin diameter, **SHD** is the shoulder diameter, **S** is the rotational speed, **F** is the feed speed, and **TA** is the tool tilt angle. The coefficients A, B, C, D, E, G, H, I, J, K, and L are determined during the modeling process.

#### Optimization process

**Figure 2:**
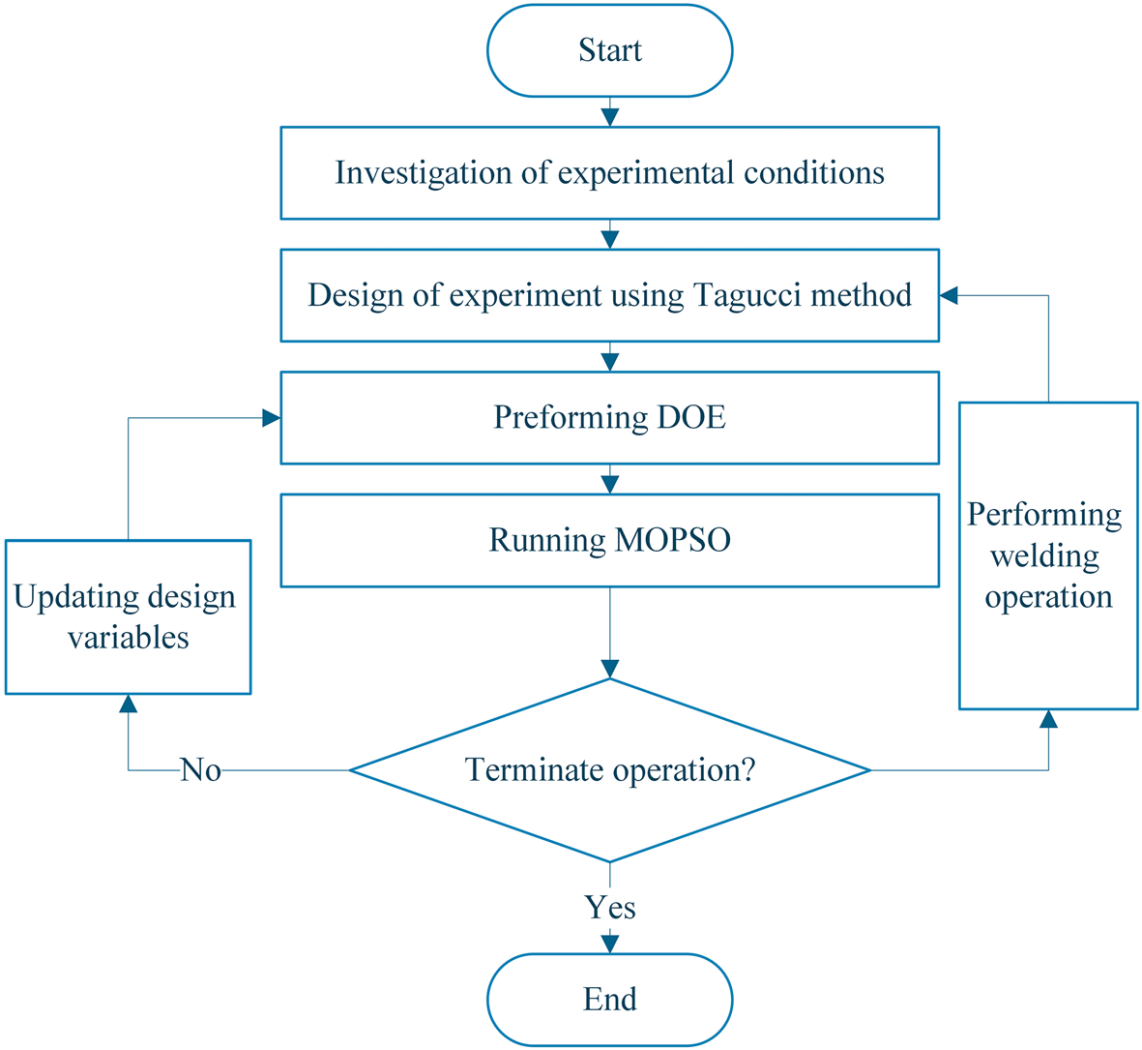
Multi-objective optimization procedure.

In this study, a MATLAB-based script is prepared to perform the multi-objective particle swarm procedure. Figure 2 shows a flowchart for the optimization process divided into the following six steps:

- *Step 1* Investigation of Experimental Condition, determining the process parameters, setting up the toolsets.
- *Step 2* Design of Experiment: Construction of Taguchi Design based on process parameters and boundaries.
- *Step 3* Performing DOE: based on the Taguchi design matrix, welding operations are performed and the objective functions are evaluated for each matrix point.
- *Step 4* Constructing RSM: according to Eq. (1), the RS functions can be constructed based on the DOE results.
- *Step 5* Running MOPSO: once the RS is constructed, the MOPSO optimization technique can search for the Pareto optimal solutions. The optimization procedure does need to perform welding but uses the RSM to replace the experiments to evaluate the value of the objective functions.
- *Step 6* Checking termination condition: The optimization procedure is terminated if the number of termination generations is satisfied. If not, the process returns to step 3, the new RS is constructed by adding new data to the design matrix.

#### Multi-objective optimization algorithm

Multi-objective optimization involves the simultaneous optimization of several objectives. To achieve this, a Pareto front solution is used^18^. For a minimization problem, point $F^{1} \left( {\mathbf{x}} \right)$ dominates point $F^{2} \left( {\mathbf{x}} \right)$ if and only if:

3 $$f_{i}^{1} \left( {\mathbf{x}} \right) \le f_{i}^{2} \left( {\mathbf{x}} \right) i = 1 \le 2$$

and for at least one j, 1 ≤ j ≤ 2, satisfying

4 $$f_{j}^{1} \left( {\mathbf{x}} \right) < f_{j}^{2} \left( {\mathbf{x}} \right)$$

That is, $F^{1} \left( {\mathbf{x}} \right)$ is a Pareto solution if it is not worse than $F^{2} \left( {\mathbf{x}} \right)$ in each of the objectives and better than $F^{2} \left( {\mathbf{x}} \right)$ in at least one of the objectives.

#### Particle swarm optimization algorithm

PSO is an evolutionary optimization technique due to the use of algorithm with only basic computational operators. Hence the implementation of this algorithm is simple and cost-effective^19,20^.

**Figure 3:**
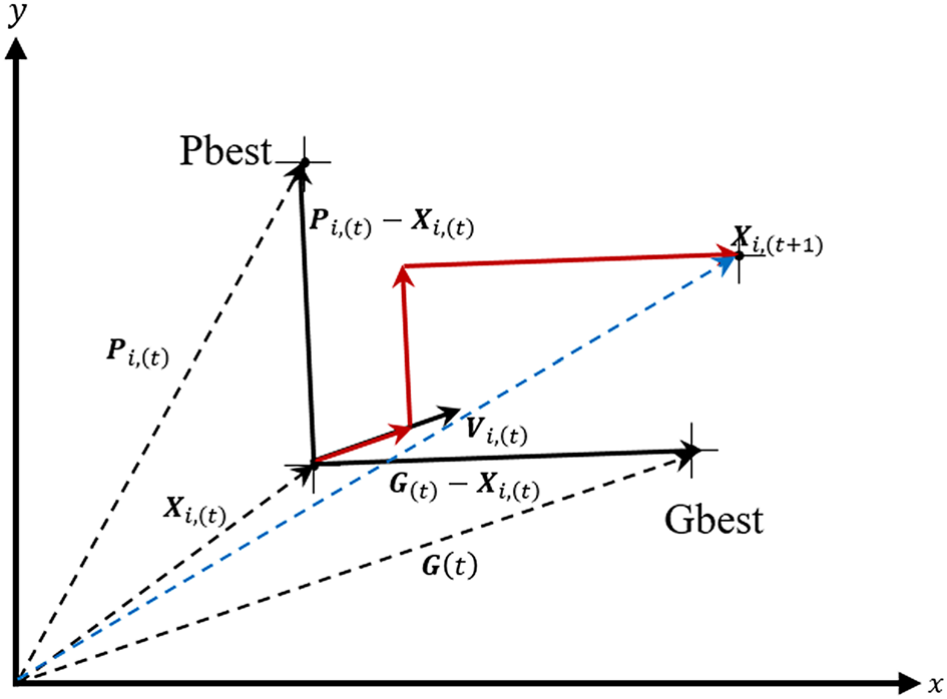
PSO algorithm performance.

Figure 3 shows the working principle of PSO algorithm. Each particle in the group can be represented by a position vector and velocity vector in a particular problem. Changing the position of each particle is possible by changing the previous position structure and velocity. Each particle carries information containing current position (*X*_*i*,(*t* )_), personal best (Pbest, best fitness that it has ever achieved in the past iterations), and Global Best (Gbest, the best fitness ever obtained by the entire group)^21^.

Each particle changes its position to obtain the best answer using the current position (*X*_*i*,(*t*)_), current velocity (*V*_*i*,(*t*)_), the distance between the current and personal best positions, and the distance between the current position and the global best position. Therefore, the new velocity vector (*V*_*i*,(*t*+*1*)_) for particle i is calculated based on the following equation:

5 $$V_{{i,\left( {t + 1} \right)}} = wV_{i,\left( t \right)} + C_{1} r_{1} \left( {P_{i,\left( t \right)} - X_{i,\left( t \right)} } \right) + C_{2} r_{2} \left( {G_{\left( t \right)} - X_{i,\left( t \right)} } \right)$$

where *w* is the inertia weight parameter. *r*_1_ and *r*_2_ are the random vectors between 0 and 1, used in maintaining group diversity. *C*_1_ and *C*_2_ are the cognitive and social parameters (acceleration parameters), respectively. Selecting the appropriate value for these parameters accelerates the convergence of the algorithm and prevents premature convergence in the local optima. The new position of the particle is obtained from Eq. (6).

6 $$X_{{i,\left( {t + 1} \right)}} = X_{i,\left( t \right)} + V_{{i,\left( {t + 1} \right)}}$$

**Figure 4:**
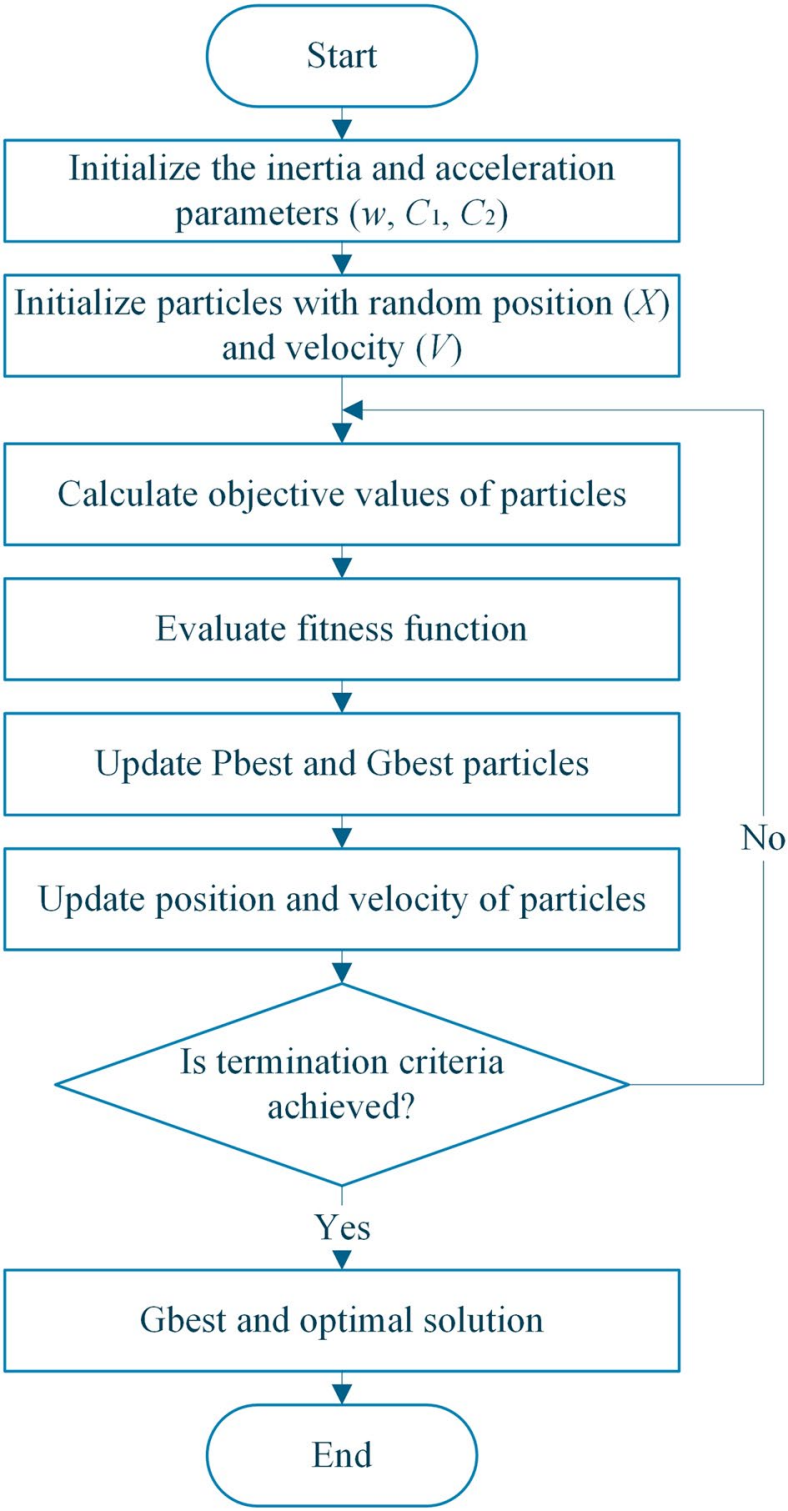
PSO flowchart.

The PSO flowchart is also shown in Fig. 4. In every iteration, after updating the position of the particles, objective functions are calculated. Then, the fitness function is evaluated to update the Pbest and Gbest. After that, the positions and velocity of particles are updated until the termination criteria is achieved.

#### Multi-objective particle swarm optimization algorithm

**Figure 5:**
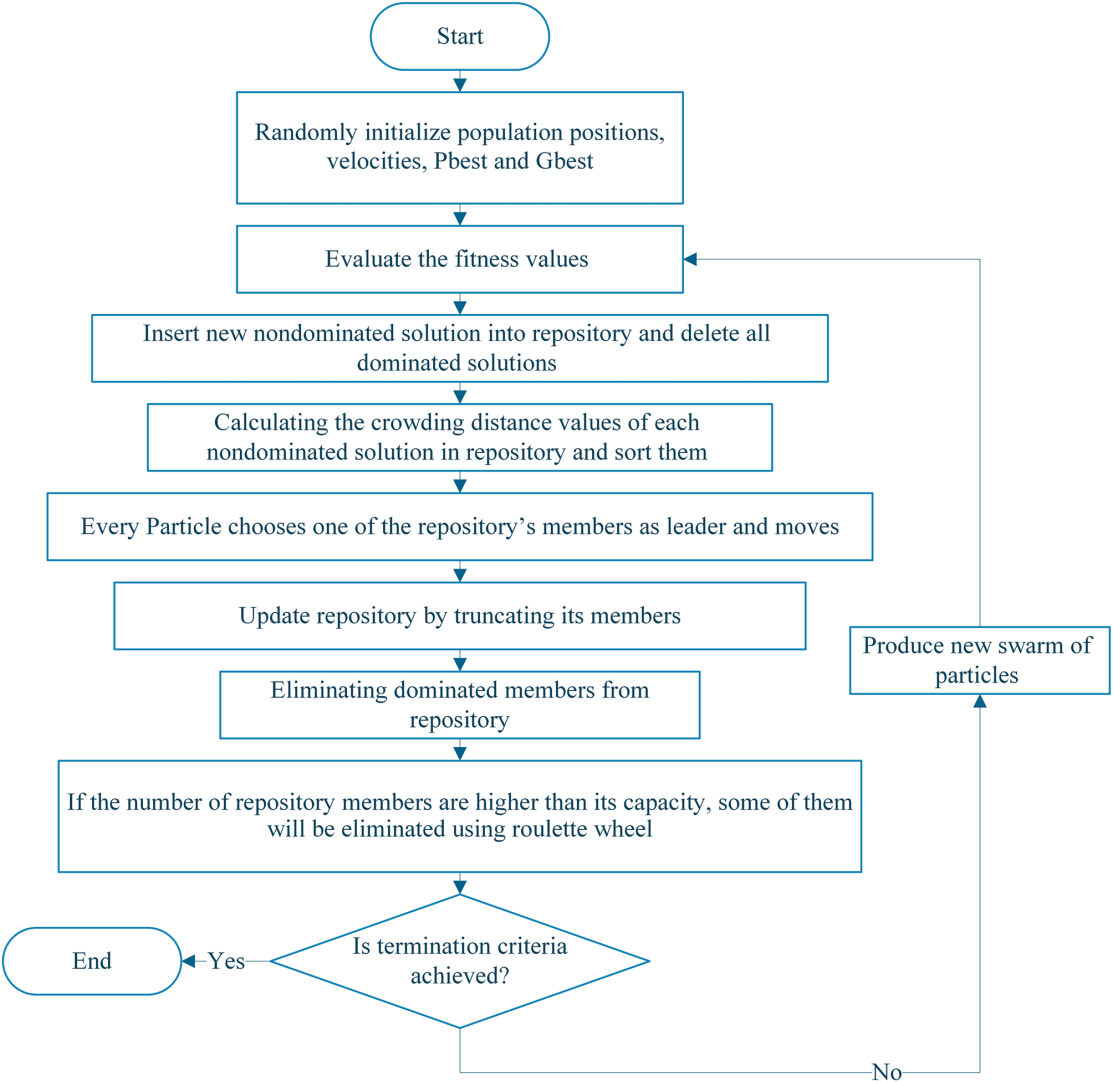
The MOPSO flowchart.

MOPSO is a generalization of the PSO algorithm used to solve multi-objective problems^22^. In the MOPSO algorithm, a concept called archive or repository (also known as the Hall of Fame) is added to the PSO algorithm. The MOPSO flowchart is shown in Fig. 5, which has the following steps:

1. Determining the parameters required for implementing the algorithm (MOPSO).
2. The initial population is created.
3. The best personal memory of each particle is determined.
4. The undominated members of the population are stored in the repository and sorted based on their crowding distance.
5. Each particle is selected a leader from among the repository members and performed its movement.
6. The best personal memories of each particle are updated.
7. New undominated members are added to the repository.
8. The dominated members of the repository are removed. If the termination conditions are not met, the algorithm is repeated from 5 onwards.

The following considerations can be made to determine the best vector of personal best memory:

1. If the new personal best dominates the best personal memory, then the new personal best replaces the current personal best memory.
2. If the new personal best is dominated by the best personal memory, then nothing will be done.
3. If neither of them dominates each other, then one is randomly considered as the best position vector.

#### Design of experiments (DOE)

Different experimental design methods are studied based on the degree of effective parameters as well as the number of effective parameters and responses. Taguchi experimental design method was employed for the DOE (Table 1).

**Table 1:** Taguchi design of experiment parameters (Specimens labeled as c and b for conical and threaded cylindrical pins, respectively).

Code		Pin diameter (mm)	Shoulder diameter (mm)	Feed speed (mm/min)	Rotational speed (rpm)	Tilt angle (°)
c1	b1	4.0	14.0	31.5	630	3.0
c2	b2	4.0	18.0	80.0	1000	3.0
c3	b3	5.0	14.0	50.0	1000	3.0
c4	b4	5.0	18.0	31.5	800	3.0
c5	b5	6.0	14.0	80.0	800	3.0
c6	b6	6.0	18.0	50.0	630	3.0
c7	b7	4.0	14.0	31.5	630	3.5
c8	b8	4.0	16.0	50.0	800	3.5
c9	b9	5.0	14.0	50.0	1000	3.5
c10	b10	5.0	16.0	80.0	630	3.5
c11	b11	6.0	14.0	80.0	800	3.5
c12	b12	6.0	16.0	31.5	1000	3.5
c13	b13	4.0	16.0	50.0	800	2.5
c14	b14	4.0	18.0	80.0	800	2.5
c15	b15	5.0	16.0	80.0	630	2.5
c16	b16	5.0	18.0	31.5	800	2.5
c17	b17	6.0	16.0	31.5	1000	2.5
c18	b18	6.0	18.0	50.0	630	2.5

### Materials

The used sheets were made of A-H121050 aluminum alloy with 5 mm thickness. The chemical composition and the base mechanical properties of this alloy are provided in Tables 2 and 3, respectively. Samples with dimensions of 50 × 50 mm were milled for welding and their surfaced were cleaned with acetone before the welding process.

**Table 2:** Chemical composition of A-H121050 aluminum alloy.

No	Element	Weight percent
1	Aluminum	Base
2	Iron	0.40
3	Copper	0.05
4	Magnesium	0.05
5	Manganese	0.05
6	Silicon	0.25
7	Titanium	0.05
8	Zinc	0.07

**Table 3:** Mechanical properties of A-H121050 aluminum.

Property	Value
Brinell hardness (HRB)	28
Shear strength (MPa)	58
Ultimate strength (MPa)	100
Yield strength (MPa)	70

### Experiments

#### Welding tools and equipment

Hot work steel H13 was used to fabricate the tools with diameters of 14, 16, and 18 mm, as it is the best material for welding aluminum parts^23^. Based on the studies performed on the welding of aluminum alloys, the most common shapes of the pin are threaded cylindrical and conical^24^.

**Figure 6:**
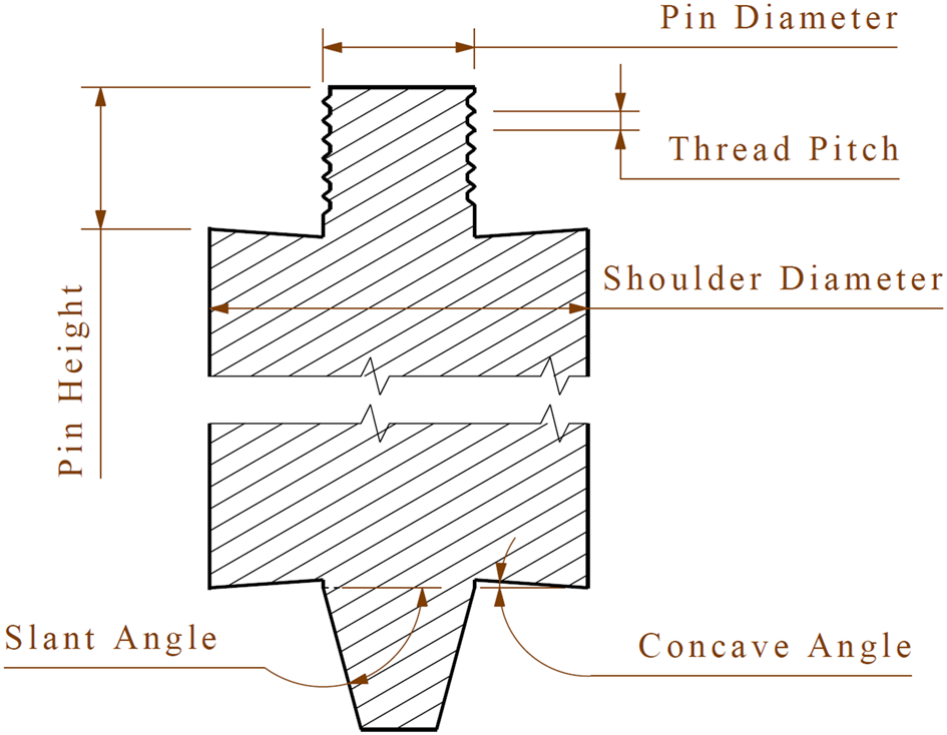
Schematic of the double-sided friction stir welding tool.

The required tools are fabricated double-sided (one side of the threaded cylinder and the other side of a simple cone) to investigate the thread effects. All shoulders were prepared with a concave angle of 5°^25^ to obtain a more functional joint. In addition, the slant angle of the conical pin was considered 75°. Figure 6 shows a schematic view of the fabricated tools. After fabrication, the tools were hardened by heat treatment up to an HRC (Hardness Rockwell C) of 50. Table 4 lists the characteristics of the tools utilized in this study.

**Table 4:** The characteristics of the tools.

Pin type	Pin diameter (mm)	Shoulder diameter (mm)	Pitch (mm)	Concave degree (°)	Pin height (mm)
Threaded cylindrical	4.0	14–16–18	0.7	5.0	4.7
	5.0	14–16–18	0.8		
	6.0	14–16–18	1.0		
Conical	4.0	14–16–18	–		
	5.0	14–16–18	–		
	6.0	14–16–18	–		

#### Tensile test

The fabricated specimens are provided from the welded parts for tensile test using a 15-ton DeghatAzma universal machine at room temperature. The cross-head velocity is adjusted 2 mm/min to satisfy the quasi-static condition. The specimen geometry all utilized relations are considered in accordance with ASTM E8. Tensile tests are conducted up to specimen failure to determine the elastic behavior and plastic and failure properties.

#### Hardness measurement

The specimens hardness at the points located in the heat-affected zones (HAZs) on the advancing and retreating sides (HAZ Adv. and HAZ Ret.) was measured using a Shegarf Abzar hardness testing apparatus based on Rockwell B (1.16-inch diameter steel ball hardness tester). The tests were conducted by applying a 100 kgf force in 10 s in accordance with ASTM-E18.

#### Impact test

**Figure 7:**
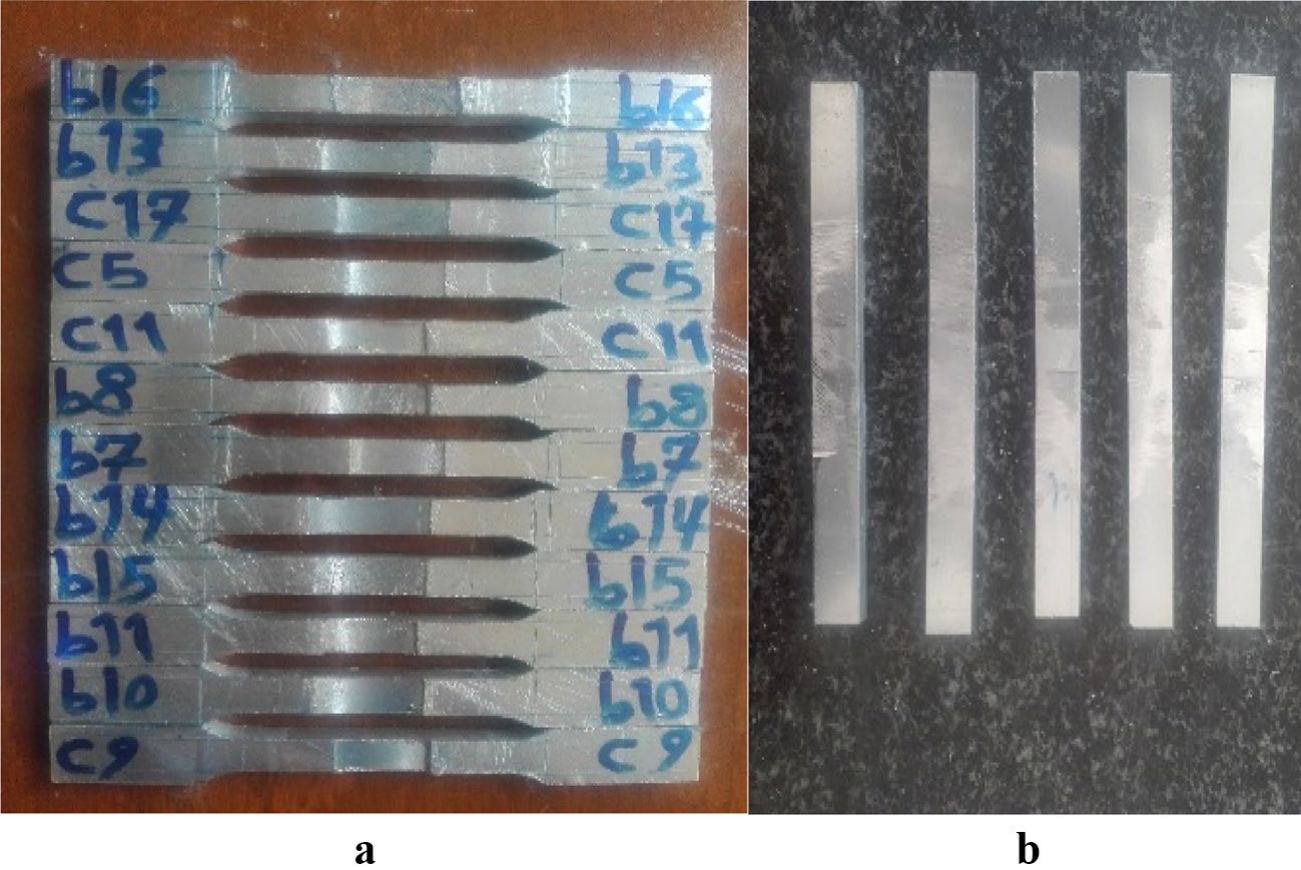
Prepared samples for (**a**) tensile test and (**b**) impact test.

The specimen required for the impact test was prepared according to ASTM E23 with a V-notch. The impact properties were evaluated using a 200 J Santam Charpy impact test machine. The specimens were carefully placed on the support by a special tool to ensure that the V-notch was in the right place related to the impact hammer. The welded specimens are prepared for tensile and impact tests by using the wire-cut machine as shown in Fig. 7.

#### Microstructural tests

The samples were fabricated in accordance with ASTME3-01. The specimens were mounted and sealed and polished using 200 to 5000 sandpapers. The samples were then etched with a 2.5% chlorine solution (HF + HCl + HNO_3_ + H_2_O). Subsequently, the transverse cross-sections of the parts were analyzed using scanning electron microscopy (SEM).

## Results

**Figure 8:**
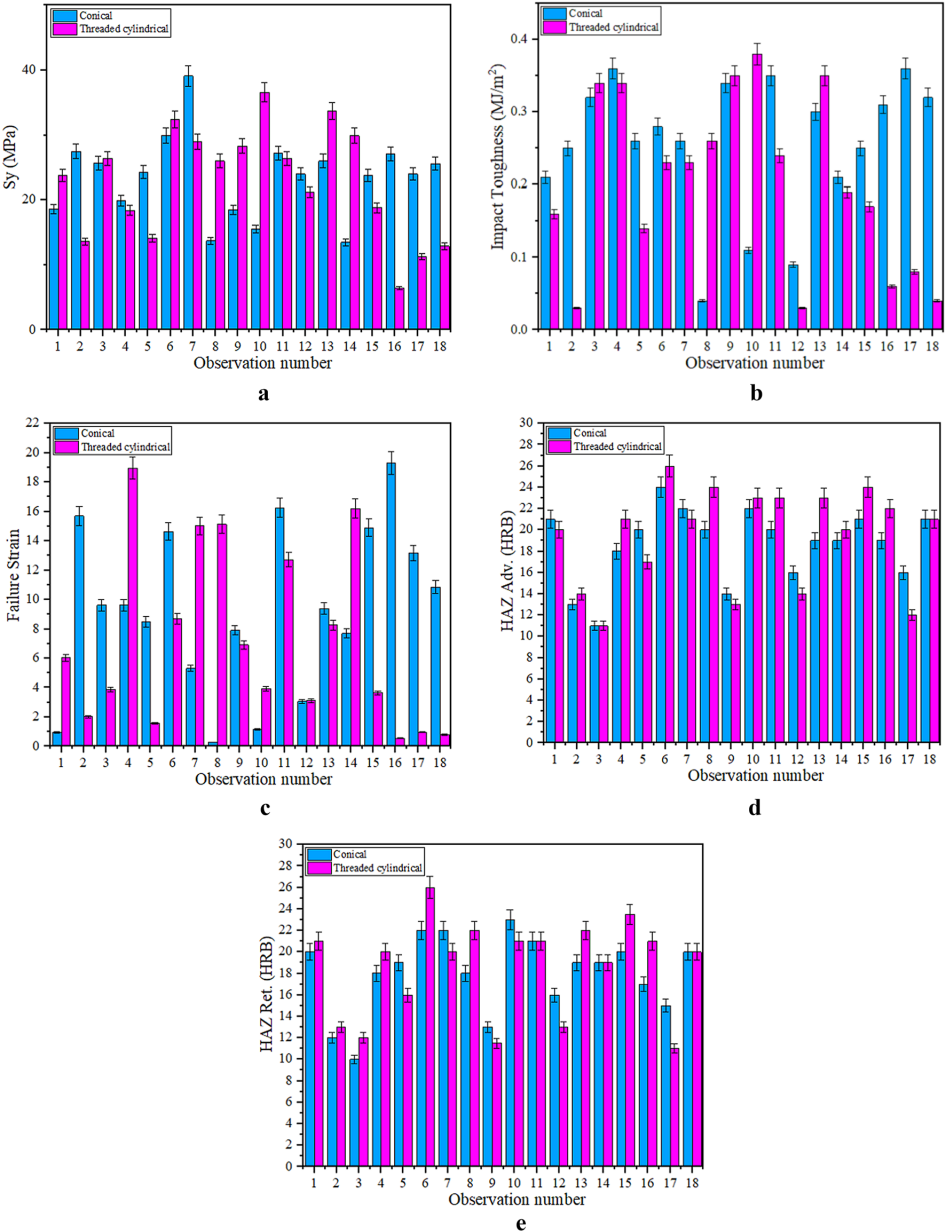
Results of the tensile test and hardness measurement of welded specimens with threaded cylindrical and conical pins: (**a**) yield strength, (**b**) impact toughness, (**c**) failure strain, (**d**) hardness on HAZ adv., and (**e**) hardness on HAZ Ret.

Figure 8 shows the complete results of the tensile test and hardness measurement of the welded specimens with threaded cylindrical and conical pins. As the figure shows, 18 points were experimentally studied and the results are given as a bar chart for yield stress, impact toughness, failure strain and material hardness. It is worth noting that the mean value of each property is reported in the figure with the related error bar.

### Response surface models

The response surface models of the objective functions were obtained as quadratic polynomials. Tables 5 and 6 show the quadratic model coefficients of the response level for the objective functions for the conical and threaded cylindrical pins, respectively, based on Eq. (2).

**Table 5:** The quadratic coefficients of response surface for the objective functions of conical pin.

Obj.\coefficient	Sy	Impact toughness	Failure strain	HAZ adv	HAZ ret
A	− 7.98	− 0.59	− 12.80	− 9.51	− 10.10
B	− 44.1	− 1.6	− 81.5	5.8	6.1
C	− 0.0579	0.0146	0.2790	− 0.2040	− 0.2950
D	0.0768	− 0.0038	− 0.0500	0.0498	0.0511
E	− 73.5	− 4.21	− 191.0	− 24.9	− 31.1
G	0.76	0.06	1.47	0.99	1.06
H	1.370	0.049	2.590	− 0.182	− 0.191
I	1.56e−3	− 1.16e−4	− 1.48e−03	1.76e−03	2.66e−03
J	− 3.96e−5	2.42e−6	3.44e−5	− 4.26e−05	− 4.39e−05
K	10.60	0.66	30.10	4.25	5.41
L	481.0	22.1	974.0	24.5	32.8

**Table 6:** The quadratic coefficients of response surface for the objective functions of threaded cylindrical pin.

Obj.\coefficient	Sy	Impact toughness	Failure strain	HAZ adv	HAZ ret
A	42.20	1.97	41.20	0.76	0.77
B	24.70	− 0.13	− 40.60	11.80	10.50
C	0.831	0.019	− 0.880	0.456	0.493
D	− 0.258	0.002	0.444	0.070	0.036
E	116.0	− 1.5	− 146.0	− 10.8	10.5
G	− 4.09	− 0.20	− 4.44	− 0.09	− 0.10
H	− 0.79	0.00	1.28	− 0.34	− 0.30
I	− 7.44e−03	− 1.70e−04	6.75e−03	− 4.15e−03	− 4.59e−03
J	1.44e−04	− 1.71e−06	− 2.73e−04	− 6.05e−05	− 3.95e−05
K	− 16.50	0.24	24.80	1.95	− 1.81
L	− 383.0	− 2.5	293.0	− 96.4	− 102.0

Table 7 lists the statistical features of the response surfaces. The response surfaces and correlation diagrams of the objective functions are presented in supplementary materials. As the table shows, the best R^2^ belongs to Hardness HAZ Adv. for both conical and threaded cylindrical pins. Since the *p* value for all objective functions is determined less than 0.05, the estimated models are found significant. In addition, the R^2^ values show an excellent prediction capability of the models in the range of 0.944–0.991 for conical yield stress and the hardness of HAZ adv. Table 8 gives the analysis of variance summary (ANOVA) of the response surface of objective functions.

**Table 7:** Statistical features of the response surfaces.

Pin type	Objective	MSE	RMSE	R^2^
Conical	Sy	2.1200	1.460	0.944
	Impact toughness	0.0004	0.021	0.975
	Failure strain	2.2300	1.490	0.966
	Hardness HAZ adv	0.1600	0.399	0.992
	Hardness HAZ ret	0.3760	0.614	0.984
Threaded cylindrical	Sy	5.2600	2.290	0.956
	Impact toughness	0.0004	0.020	0.985
	Failure strain	1.8200	1.350	0.974
	Hardness HAZ adv	0.3360	0.580	0.992
	Hardness HAZ ret	0.4060	0.637	0.990

**Table 8:** ANOVA summary.

Pin type	Objective	Sum of squares	Degree of freedom	Mean square	F value	*p* value prob > F	Status
Threaded cylindrical	Sy	978.72	10	97.872	5.815	0.033	Significant
	Impact toughness	0.19	10	0.019	12.65	0.013	Significant
	Failure strain	535.82	10	53.58	9.19	0.012	Significant
	Hard. HAZ adv	347.56	10	34.76	32.31	0.001	Significant
	Hard. HAZ ret	336.00	10	33.60	25.87	0.001	Significant
Conical	Sy	241.80	10	24.180	4.880	0.033	Significant
	Impact toughness	0.12	10	0.012	7.65	0.032	Significant
	Failure strain	503.60	10	50.36	7.04	0.022	Significant
	Hard. HAZ adv	167.17	10	16.72	36.98	0.000	Significant
	Hard. HAZ ret	196.65	10	19.67	18.42	0.001	Significant

### Effect of parameters on objective functions

**Figure 9:**
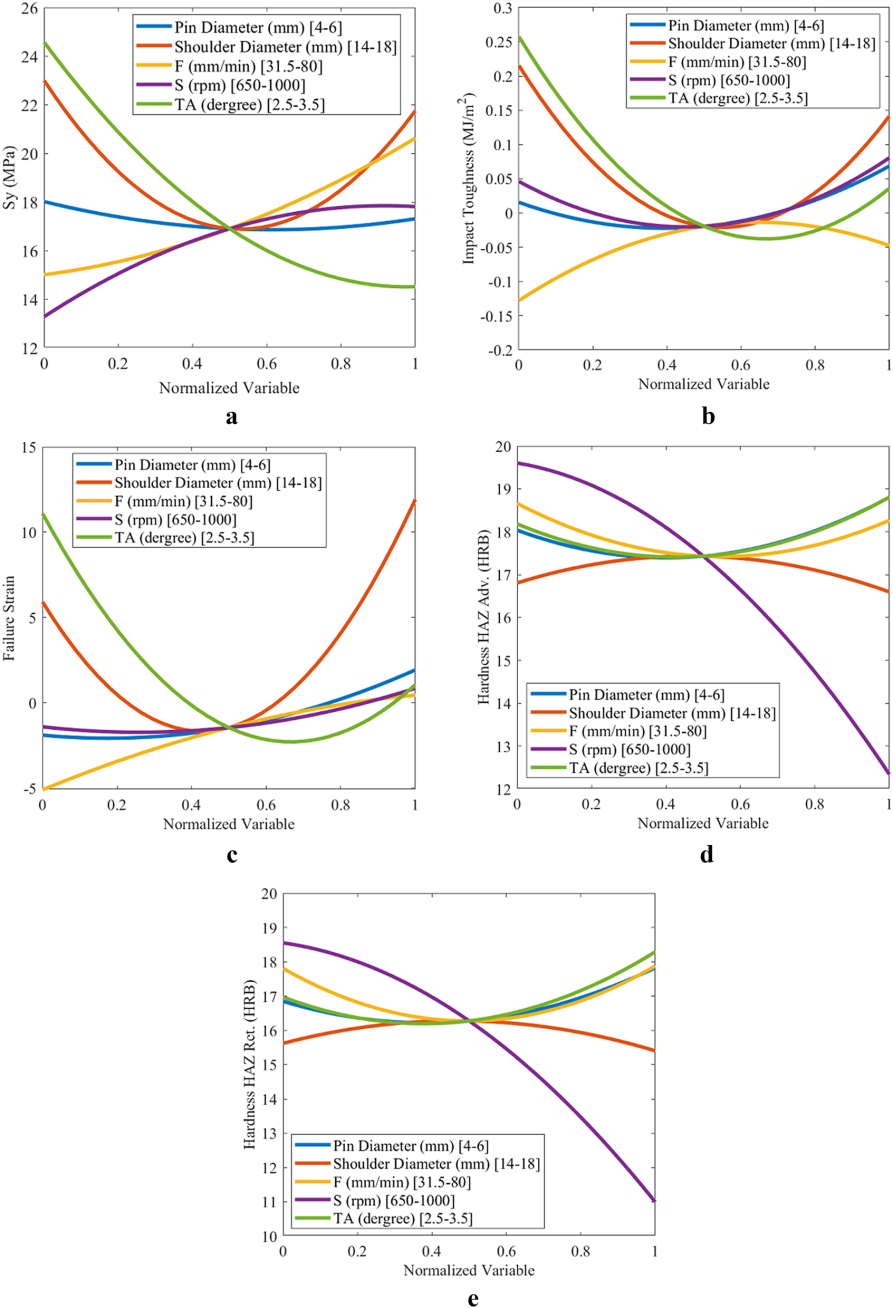
Variations in conical pin objective functions for (**a**) yield strength, (**b**) impact toughness, (**c**) failure strain, (**d**) hardness on HAZ adv., and (**e**) hardness on HAZ Ret.

**Figure 10:**
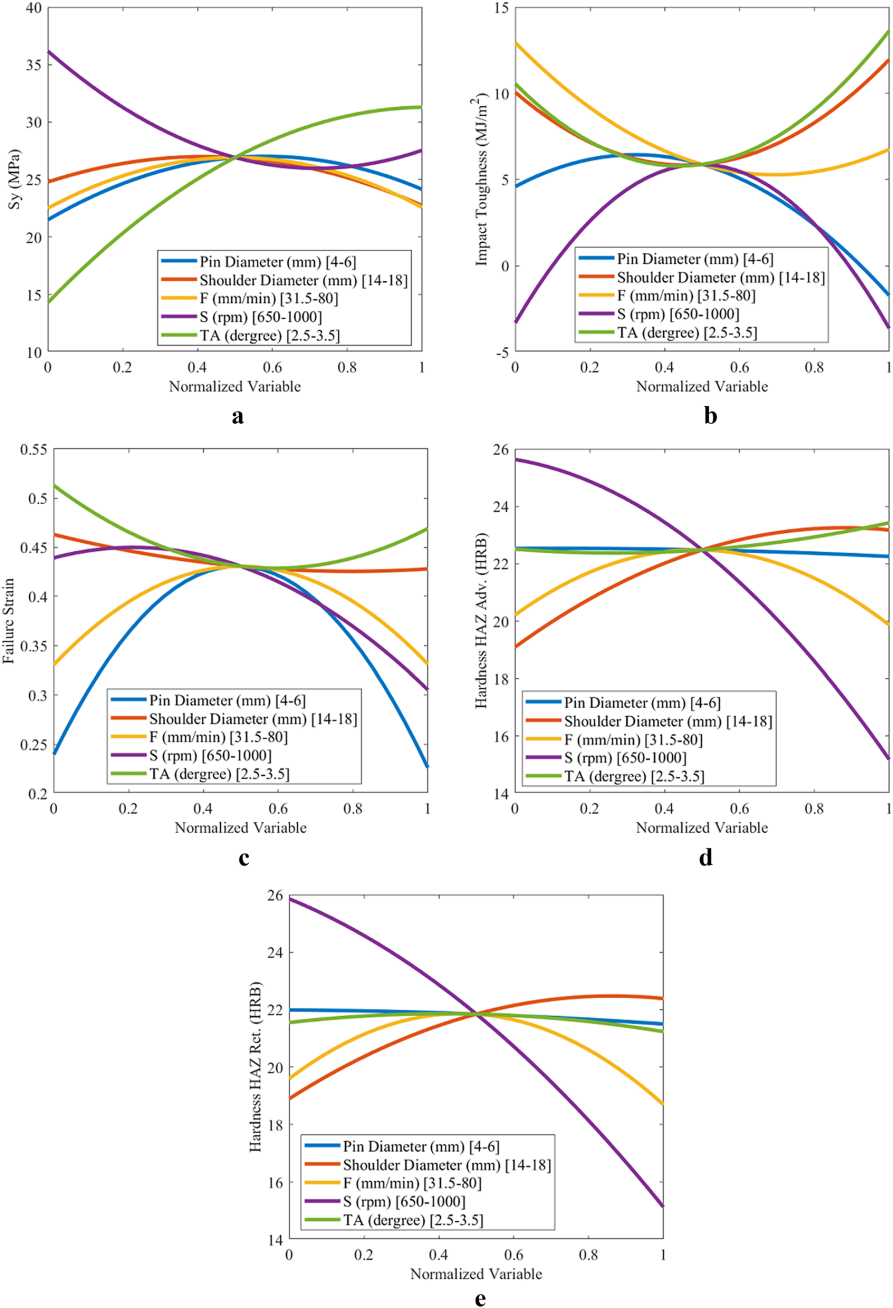
Variations in threaded cylindrical pin objective functions for (**a**) yield strength, (**b**) impact toughness, (**c**) failure strain, (**d**) hardness on HAZ adv., and (**e**) hardness on HAZ Ret.

**Figure 11:**
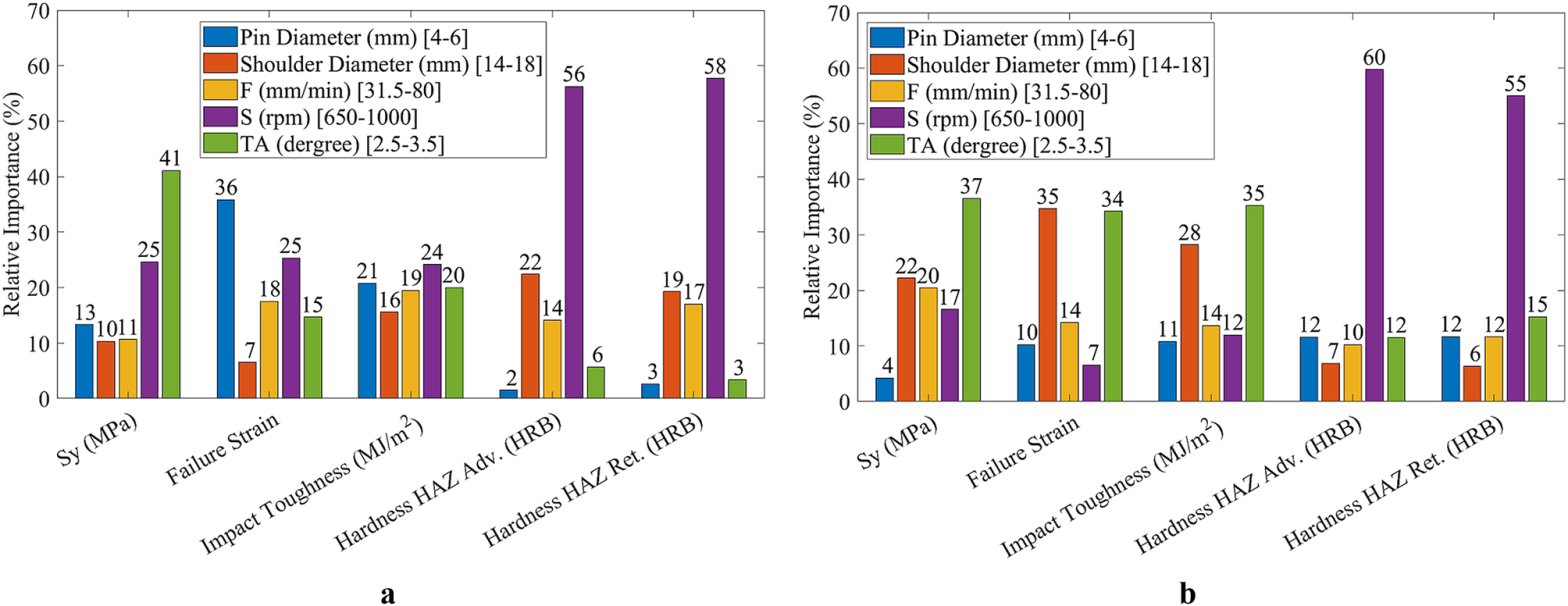
Relative importance of parameters affecting objective functions: (**a**) conical pin, (**b**) threaded cylindrical pin.

Figures 9 and 10 depict the variations in the objective functions for each parameter in the design interval for the conical and threaded cylindrical pin, respectively. All of the parameters are normalized in the range of 0–1 based on their boundaries to plot these diagrams. The objective function is plotted by variation of every single parameter, while the other four parameters are fixed on the normalized value of 0.5. Figure 11 illustrates the relative importance of the parameters based on objective function variation between boundaries to better understand how parameters affect the objective functions.

As shown in Fig. 11a, the most important parameter for hardness HAZ adv. is S = 60%. However, this parameter is determined to be the least relevant for impact toughness by only 7%. As illustrated in the figure, S shows a significant effect on both Adv. and Ret. harnesses. In contrast, the shoulder diameter controls the impact toughness, yield strength, and failure strain by 35%, 22%, and 28%, respectively.

The order of the effect of the process parameters on the objective functions of the conical pin is given as follows:

1. *Yield strength* shoulder diameter, tilt angle, feed speed, rotational speed, and pin diameter.
2. *Impact toughness* tilt angle, shoulder diameter, feed speed, rotational speed, and pin diameter.
3. *Failure strain* shoulder diameter, tilt angle, feed speed, rotational speed, and pin diameter.
4. *Hardness of HAZ Adv.* rotational speed, pin diameter and tilt angle, feed speed, and shoulder diameter.
5. *Hardness of HAZ Ret.* rotational speed, tilt angle, pin diameter, feed speed, and shoulder diameter.

The order of the effect of the process parameters on the objective functions of the threaded cylindrical pin is listed as follows:

1. *Yield strength* tilt angle, rotational speed, pin diameter, feed speed, and shoulder diameter.
2. *Impact toughness* pin diameter, rotational speed, feed speed, tilt angle, and shoulder diameter.
3. *Failure strain* rotational speed, pin diameter, tilt angle, feed speed, and shoulder diameter.
4. *Hardness of HAZ Adv.* rotational speed, shoulder diameter, feed speed, tilt angle, and pin diameter.
5. *Hardness of HAZ Ret.* rotational speed, shoulder diameter, feed speed, tilt angle, and pin diameter.

In this research, MATLAB Model-Based Calibration Toolbox is used to perform RSM, and a MATLAB-based script is developed for ANOVA.

### Optimization results

**Figure 12:**
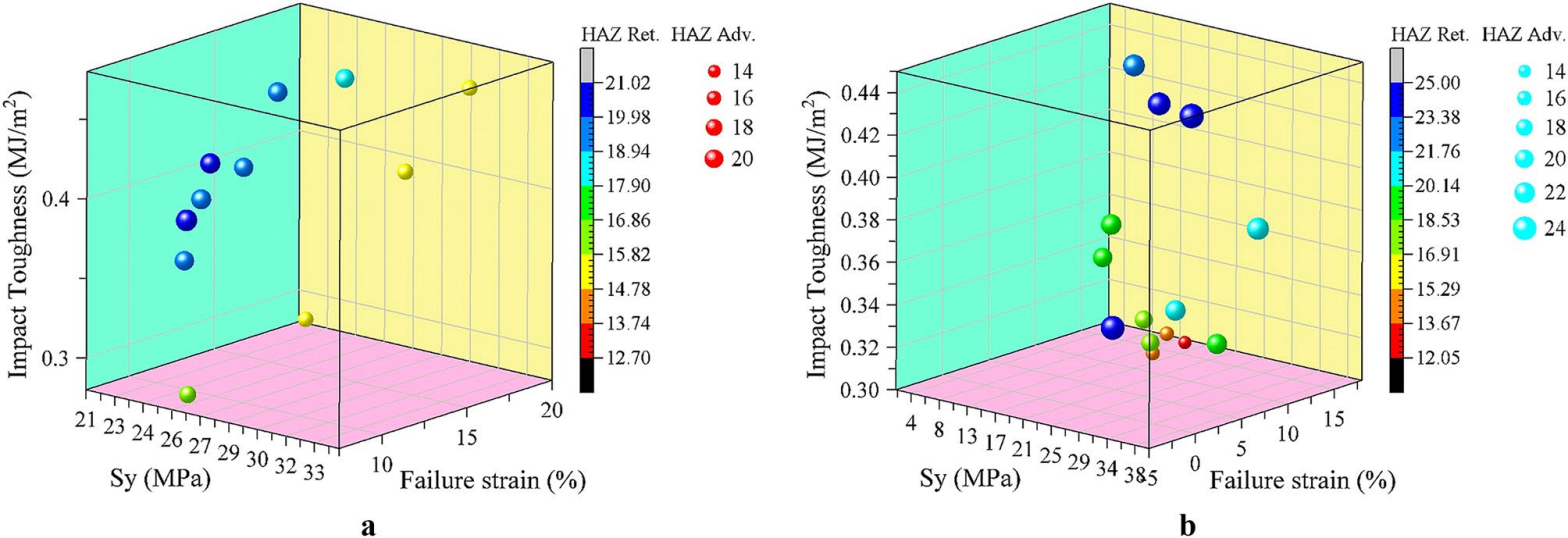
Multi-objective optimization Pareto front: (**a**) conical pin, (**b**) threaded cylindrical pin.

The optimization process is performed using the experimental data based on the multi-objective particle swarm method. Figure 12 demonstrates the Pareto front obtained for both conical and threaded cylindrical pins.

**Figure 13:**
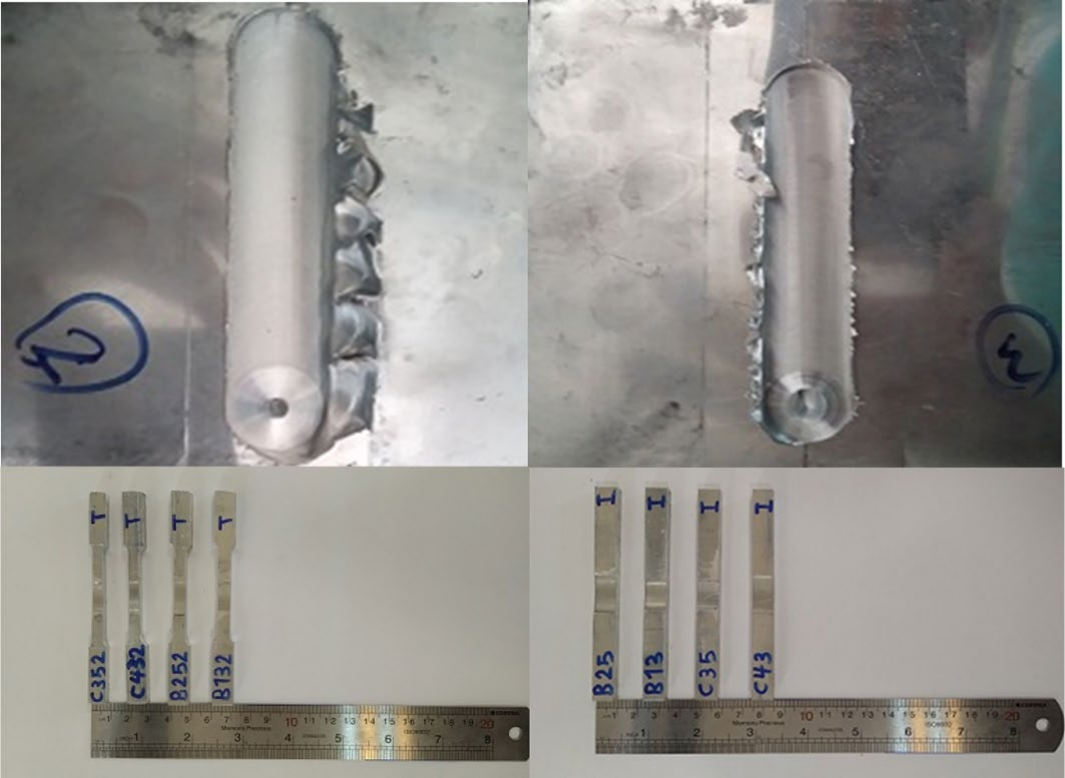
The welded specimens with the optimized welding values.

In Fig. 12 every point can be considered optimal, depending on the priority of the objective function; if an equal priority is required, the closest point to the origin can be selected as the optimal design. In this study, for each tool type, a point is selected for the experiment, two samples are welded with optimization solutions, and the objective functions are obtained using tensile, impact, and hardness tests (Fig. 13).

Table 9 presents the results of the experiments. As Table 9 suggests, the model accuracy in predicting objective functions with optimal values is excellent in most cases, indicating the reliability of the proposed model.

**Table 9:** The mechanical properties of samples fabricated with the optimal welding values.

Parameter/objective	Threaded cylindrical pin	Conical pin
Pin diameter (mm)	4.49	4.50
Shoulder diameter (mm)	16.62	14.03
Feed rate (mm/min)	41.23	67.66
Rotational speed (rpm)	814.72	867.73
Tool tilt angle (°)	3.41	2.54
**Predicted yield stress (MPa)**	**27.15**	**32.15**
Experimental yield stress (MPa)	31.04	27.60
*Yield stress prediction error (%)*	*14.34*	*14.14*
**Predicted impact toughness (MJ/m**^**2**^**)**	**0.47**	**0.37**
Experimental impact toughness (MJ/m^2^)	0.44	0.36
*Impact toughness prediction error (%)*	*5.64*	*1.36*
**Predicted failure strain**	**15.91**	**17.51**
Experimental failure strain	16.81	16.49
*Failure strain prediction error (%)*	*5.69*	*5.81*
**Predicted HAZ hardness on advancing side (HRB)**	**22.98**	**16.48**
Experimental HAZ hardness on advancing side (HRB)	24.56	14.90
*HAZ hardness on advancing side prediction error (%)*	*6.88*	*9.58*
**Predicted HAZ hardness on retreating side (HRB)**	**21.24**	**15.38**
Experimental HAZ hardness on retreating side (HRB)	22.34	14.20
*HAZ hardness on retreating side prediction error (%)*	*5.17*	*7.67*

### Metallography

Two low-quality (b17 and c12) and two optimal samples are compared using microstructural tests to evaluate the weld quality. Excessive heat causes a coarse-grained structure and reduces the weld strength. The grain shape and size are the functions of material heat and flow. Due to the frictional contact between shoulder and work surface, heat is generated during the rotation of the tool, and the material undergoes a severe deformation at high temperature of the joint with the pin rotational movement. Thus, small and coaxial recrystallized grains are formed. The pin shape can adjust the material flow and causes fine graining. Once an optimal pin is employed, the forging forces in the welding zone are increased. This implies an improvement in material microstructures.

**Figure 14:**
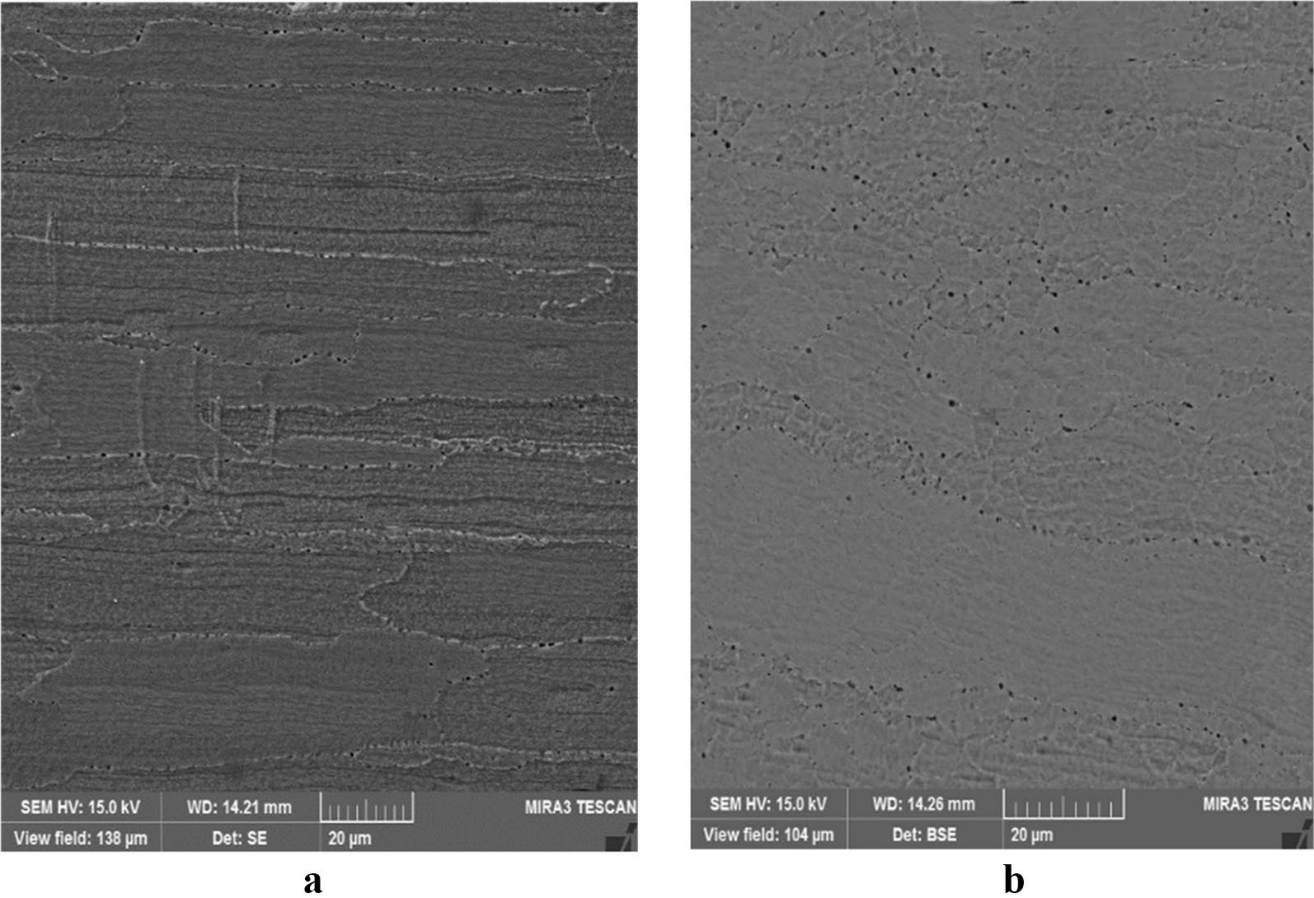
SEM images of the stir zone in the optimal samples: (**a**) conical pin, (**b**) threaded cylindrical pin.

Figure 14 shows SEM images of the stir zone in the optimal samples. In this region, the grains are coaxial and fine, indicating the occurrence of dynamic recrystallization. This can be attributed to the reduction of heat compared to weak samples during welding operations.

**Figure 15:**
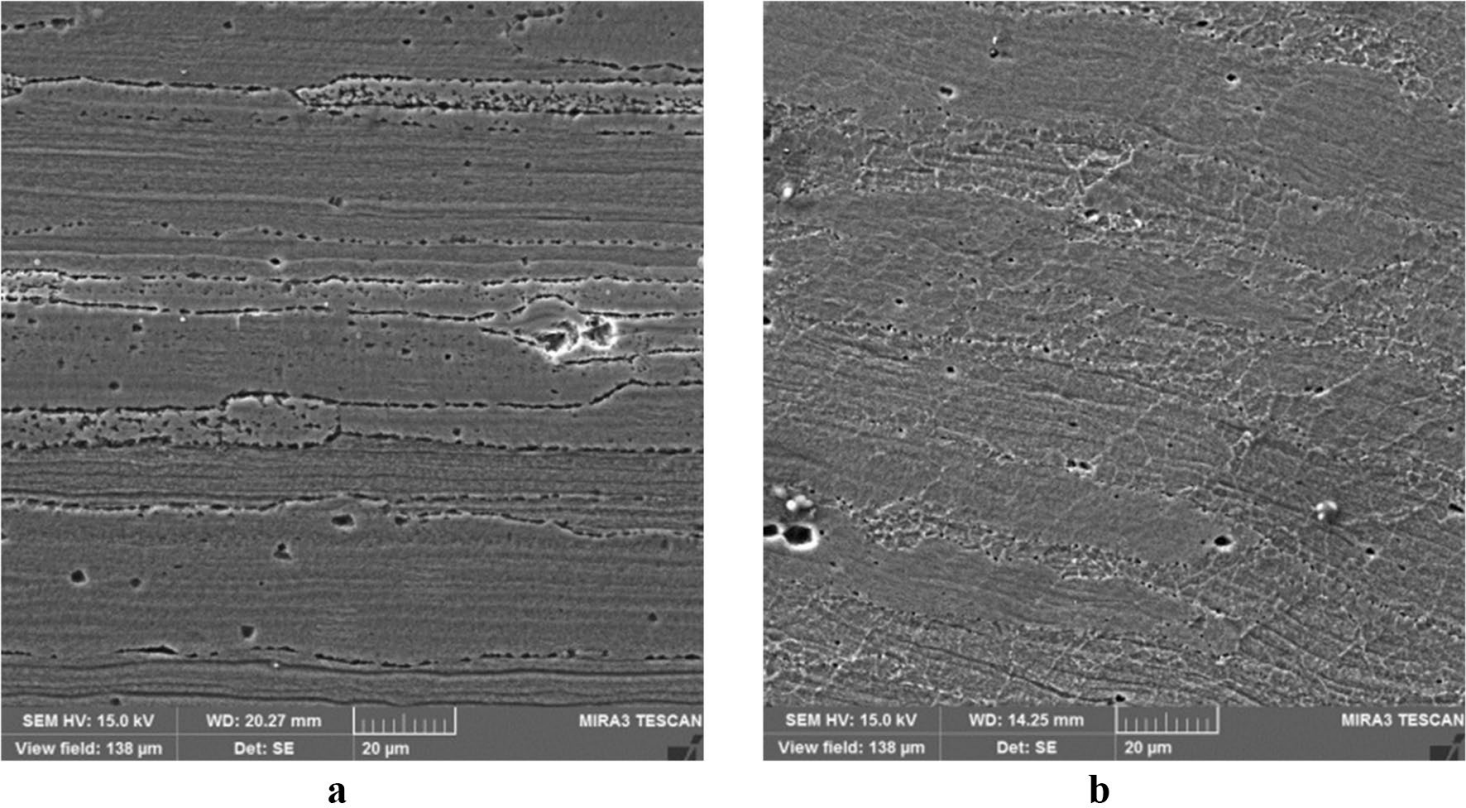
SEM images of the stir zone in the low-quality samples: (**a**) conical pin (c12), (**b**) threaded cylindrical pin (b17).

Figure 15 presents SEM images of the stir zone of the low-quality samples. In comparison with Fig. 14, it is clear that the grains are more prominent and defective. The observed holes are formed due to the high-temperature deformation and they are reduced the joint strength and hardness. Comparing the obtained experimental strength and stiffness proves the above observations.

**Figure 16:**
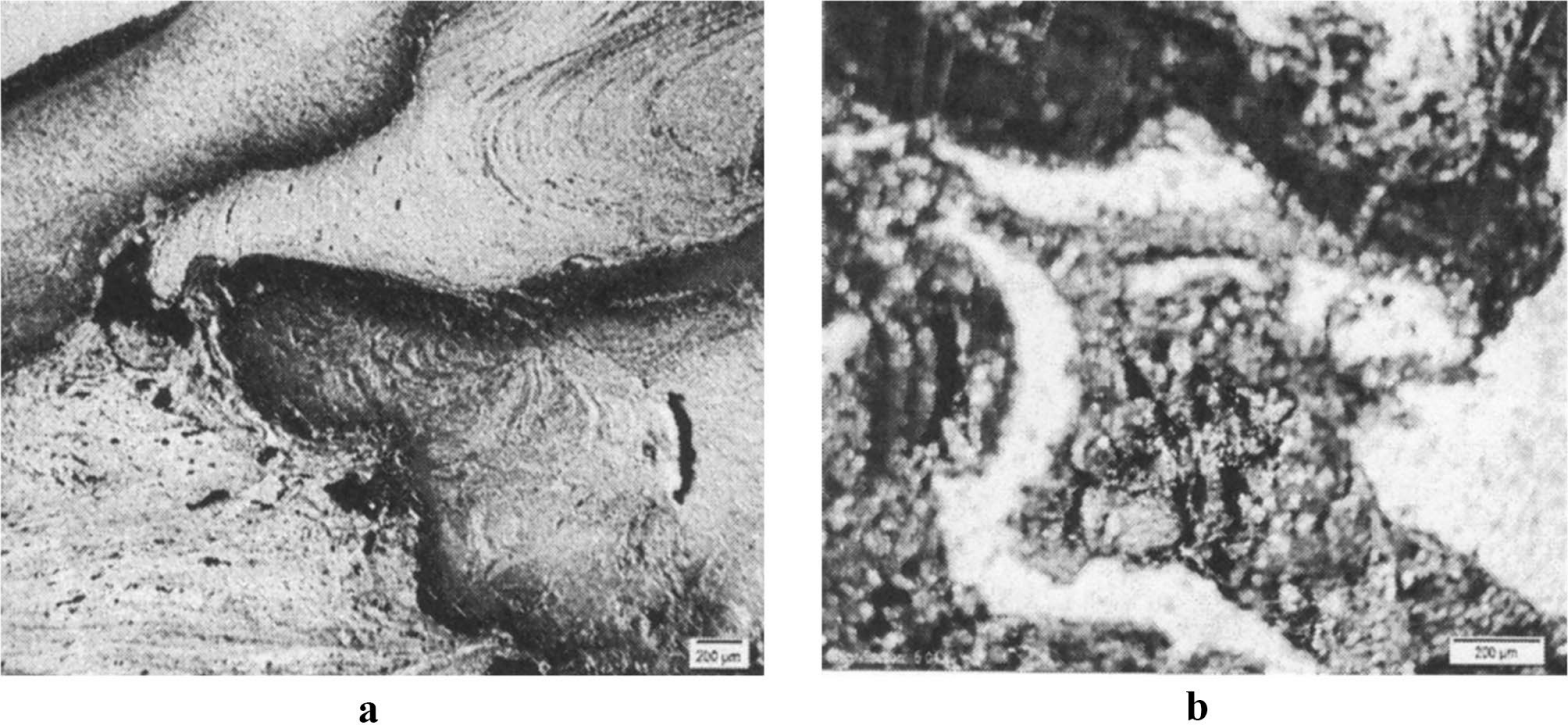
Macroscopic images of the weld cross-section of (**a**) b17 and (**b**) c12 samples.

Lower heat is generated in the optimal samples owing to low rotational speed and high feed speed. In low-quality samples, more heat is generated due to the higher rotational speed and lower feed speed. Figure 16 shows microscopic images of the weld cross-section of b17 and c12 samples. The presence of tunnel cavities is significant because the extra heat is generated in the process.

**Figure 17:**
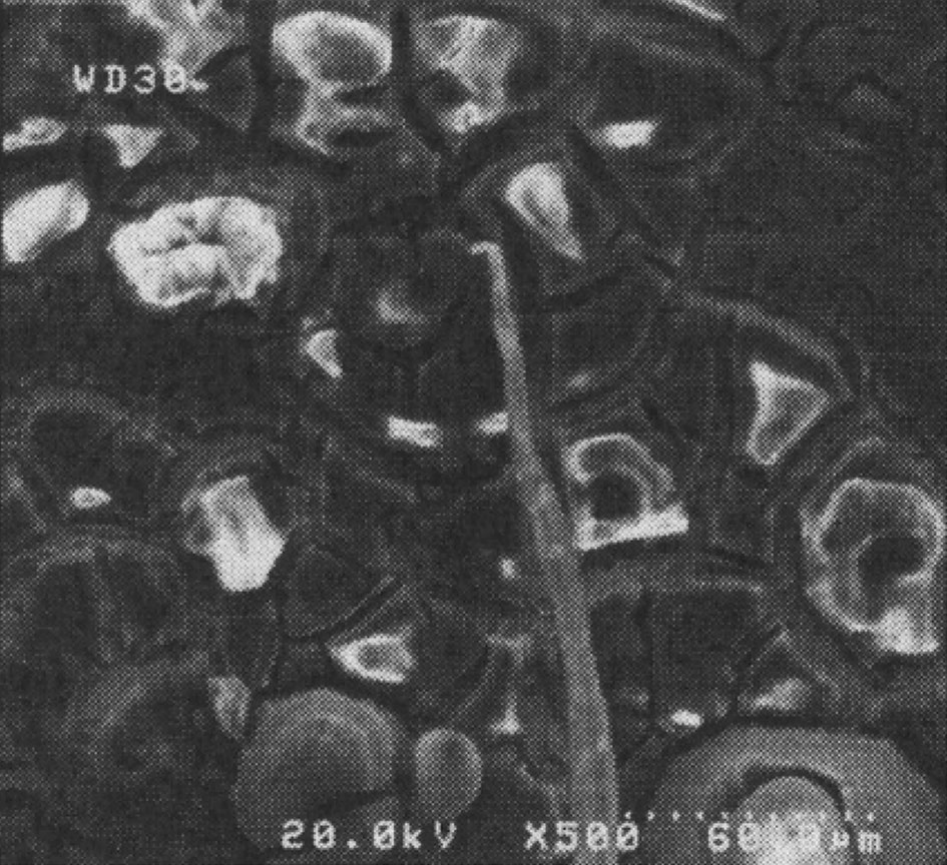
Macroscopic image of oxide layers in sample c12.

The formed holes are observable in the upper third of the weld, which is affected by the tool shoulder and forging force. Excessive heat also causes some welding layers to oxidize, as shown in Fig. 17.

**Figure 18:**
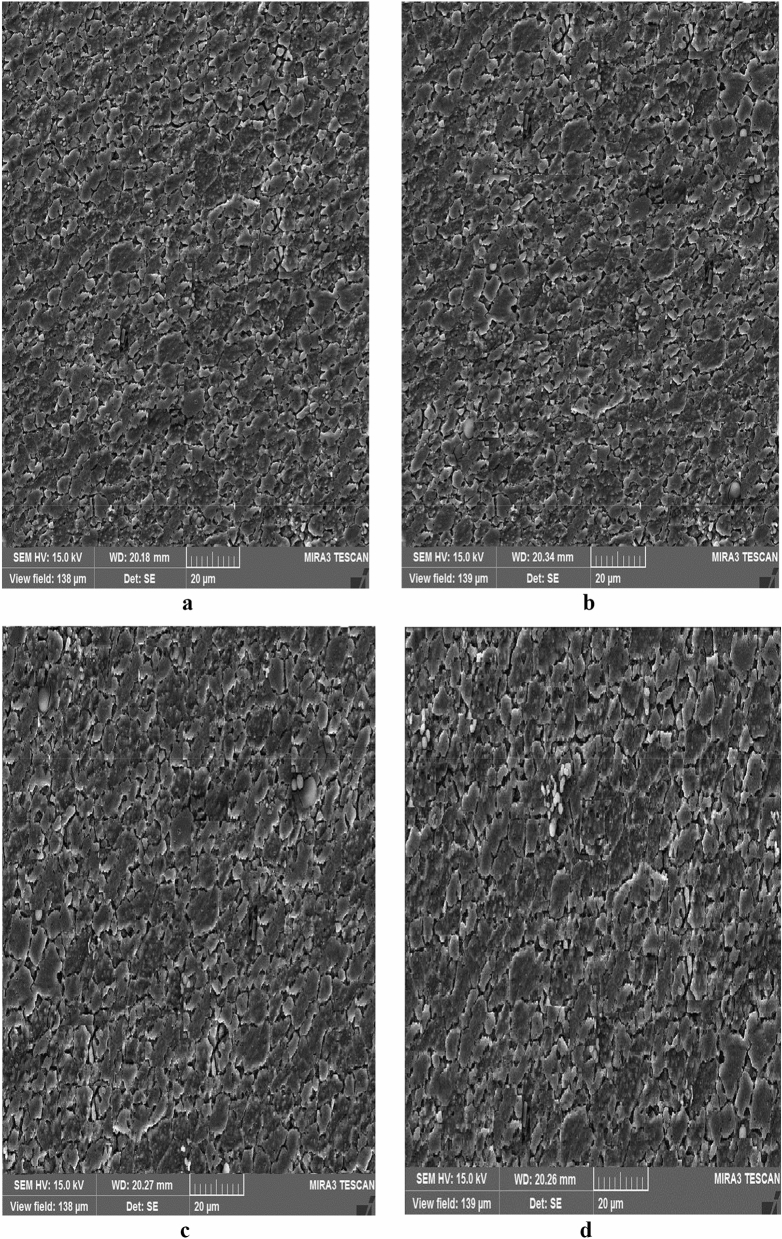
SEM images of the thermo-mechanically affected zone region of the samples: (**a**) threaded cylindrical pin, (**b**) conical pin, (**c**) b17, and (**d**) c12.

Figure 18 shows SEM images of the thermo-mechanically affected zones of the samples, and no defects and separation are seen in the intersection in the optimum parts. In both cases, acceptable welding is achieved due to sufficient heat production caused by rotational and proper feed speeds. Improvement in the material flow is observed for the threaded cylindrical pin compared with the conical pin due to the geometric shape of the tool pin. Proper penetration of particles into each other is clearly seen in these images, and this enhances the material strength and prevents crack formation in the welded specimen. As expected, no dendrite formation is detected in the stir and thermo-mechanically affected zones after the welding process. This defect type is not usually found in semi-solid materials^26,27^.

By dividing the welding section into three parts, we can see that the main stirring zone is placed in the upper third of the weld, affected by the tool shoulder. In this region, before two parts are forged, a paste state is formed in the joint parts, and the particles penetrate each other. The lower two-thirds of the weld is affected by the pin. In this area, the metal is extruded from the front to back of tool; therefore, stirring is less than one-third of the upper. As stated before, it can be concluded that fine-grained and flawless structures are occurred at lower temperatures, and coarser and weaker structures are produced at higher temperatures.

However, it should be noted that in addition to rotational and feed speeds that cause heat changes, other factors such as the tool penetration depth could also affect the heat production. For example, in b10, despite the low rotational speed and high feed speed, heat is obtained lower than others due to the lower tool penetration depth into the workpiece. Hence no appropriate mechanical connection is formed, and the lower strength and hardness values for this sample are achieved. In the c17 sample, although the high rotational speed and the low feed is utilized, due to the reduction of the penetration depth of the tool, heating is reduced to the optimal level and a joint with the desired strength and mechanical properties can be obtained.

In addition to the tool penetration depth, the tilt angle also affects the heat production and, as a result, the formation of microstructure and desirable or undesirable mechanical properties. This is evident in the comparison of the results of b6 and b18. In these two samples, increasing the tool tilt angle is reduced the mechanical properties (Fig. 7).

## Conclusion and discussion

In this study, multi-objective optimization of the objective functions including yield strength, impact toughness, fracture strain, and hardness in HAZ on the advancing and retreating in frictional stir welding of 1050A-H12 aluminum was performed using the response surface method and MOPSO method. In addition, the effects of pin and shoulder diameter, pin geometry, rotational and feed speeds, and tilt angle on the above objectives were investigated, and the following results were obtained.

1. The lower rotational speed and higher feed speed are generated lower heat; consequently, the material strength and hardness are improved.
2. Based on the metallographic analysis, the proposed model is desirable for achieving a fine-grained structure with minimum defects and optimal mechanical properties for samples fabricated at low temperatures.
3. In addition to the rotational and feed speeds, which could change the generated heat, other factors such as the tool penetration depth affect heat production. For example, in b10, despite the low rotational speed and high feed speed, heat production is low due to the low tool penetration depth into the workpiece, preventing the formation of a proper mechanical connection. Therefore, the strength and hardness values for this sample are low. In addition, in the c17 sample, although the rotational speed is high and the feed is low, it still results from the reduction of the penetration depth of the tool, heating is reduced to the optimal level, and a joint with the desired strength and mechanical properties can be observed.
4. Improvement in the material flow is observed for the threaded cylindrical pin compared with the conical pin due to the geometric shape of the tool pin leading to more functional mechanical properties.
5. Increasing the tool tilt angle is reduced the mechanical properties by comparing the results of b6 and b18.
6. The combination of the response surface methodology and the multi-objective particle swarm algorithm led to the modeling and optimization of the process with outstanding accuracy and low experimental cost.

## Supplementary Information

Supplementary Information.
