# Supplementary material for: A multi-objective optimization using response surface model coupled with particle swarm algorithm on FSW process parameters
Source: Sci Rep. 2022 Feb 18;12:2837. doi: 10.1038/s41598-022-06652-3 (PMC8857264; doi:10.1038/s41598-022-06652-3)
Supplement: Supplementary file 1 — Supplementary Information. [file 41598_2022_6652_MOESM1_ESM.docx]

**A multi-objective optimization using response surface model coupled with particle swarm algorithm on FSW process parameters**

**Parviz Kahhal**1,2***, Mohsen Ghasemi**3**, Mohammad Kashfi**2**, Hossein Ghorbani-Menghari**1**, & Ji Hoon Kim**1*

1Pusan National University, School of Mechanical Engineering, 2 Busandaehak-ro 63beon-gil, Geomjeong-gu, Busan, 46241, South Korea.

2Ayatollah Boroujerdi University, Department of Mechanical Engineering, Boroujerd, 69199-69737, Iran.

3Dezful Branch, Islamic Azad University, Mechanical Engineering Department, Dezful, 303, Iran.

*corresponding. kimjh@pusan.ac.kr, parvizkahhal@abru.ac.ir

# **Supplementary material**

Figures [S1](#FigureS1)and [S3](#FigureS3) show the response surfaces of the objective functions for the conical and threaded cylindrical pins, respectively. To plot these surfaces, two parameters are considered as x and y axes, while other three parameters are fixed in the mean value of their boundaries, z axis is the correlation diagrams of the objective functions presented in Figures [S2](#FigureS2) and [S4](#FigureS4). The x axis is the observed value of the objective functions in the experiments and y axis is predicted value via RSM.

**Figure S1.** Response surface of conical pin objectives versus design parameters: a-c) Yield strength, d-f) Impact toughness, g-i) Failure strain, j-l) Hardness on HAZ adv., and m-o) Hardness on HAZ ret.

**Figure S2.** Experimental observations versus predicted ones for conical pin: a) Yield strength, b) Impact toughness, c) Failure strain, d) Hardness on HAZ adv., and e) Hardness on HAZ Ret.

**Figure S3.** Response surface of threaded cylindrical pin objectives versus design parameters: a-c) Yield strength, d-f) Impact toughness, g-i) Failure strain, j-l) Hardness on HAZ adv., and m-o) Hardness on HAZ ret.

**Figure S4.** Experimental observations versus predicted ones for threaded cylindrical pin: a) Yield strength, b) Impact toughness, c) Failure strain, d) Hardness on HAZ adv., and e) Hardness on HAZ Ret.
